# Dietary Supplement Use during Preconception: The Australian Longitudinal Study on Women’s Health

**DOI:** 10.3390/nu9101119

**Published:** 2017-10-13

**Authors:** Elle McKenna, Alexis Hure, Anthony Perkins, Ellie Gresham

**Affiliations:** 1 Griffith Health, Griffith University, Southport, QLD 4215, Australia; a.perkins@griffith.edu.au (A.P.); ellie.gresham@health.nsw.gov.au (E.G.); 2 School of Medicine and Public Health, The University of Newcastle, Callaghan, NSW 2308, Australia; alexis.hure@newcastle.edu.au

**Keywords:** dietary supplements, preconception, multivitamins, maternal

## Abstract

Worldwide, dietary supplement use among reproductive aged women is becoming increasingly common. The aim of this study was to investigate dietary supplement use among Australian women during preconception. Self-reported data were collected prospectively for the Australian Longitudinal Study on Women’s Health (ALSWH). The sample included 485 women aged 31–36 years, with supplement data, classified as preconception when completing Survey 5 of the ALSWH in 2009. Frequency and contingency tables were calculated and Pearson’s chi-square test for associations between demographic variables and supplementation status was performed. Sixty-three per cent of women were taking at least one dietary supplement during preconception. Multiple-micronutrient supplements were the most commonly reported supplement (44%). Supplements containing folic acid and iodine were reported by 51% and 37% of preconception women, respectively. Folic acid (13%), omega-3 fatty acids (11%), vitamin C (7%), B vitamins (4%), iron (3%), and calcium (3%) were the most common single nutrients supplemented during preconception. Women trying to conceive, with no previous children, and born outside Australia were more likely to take dietary supplements. In Australia, dietary supplement use during preconception is relatively high. However, supplementation of recommended nutrients, including folic acid and iodine, could be improved.

## 1. Introduction

Worldwide, women of reproductive age are routinely recommended nutrient supplementation during preconception and pregnancy to reduce their risk of adverse pregnancy and foetal outcomes associated with nutrient deficiencies [1,2,3]. The effectiveness of folic acid supplementation in the prevention of Neural Tube Defects (NTDs) is well documented [4,5]. The neural tube develops during the first few weeks of pregnancy [6], which is often before a woman recognises she is pregnant and well before her first antenatal care appointment [2]. Following years of voluntary folic acid fortification, mandatory fortification of wheat flour used for bread-making with folic acid was implemented in Australia in September 2009. The prevalence of folic acid deficiency in Australia has reduced, and there has also been a significant decline of 14.4% in the rate of NTDs per 10,000 conceptions, by March 2011 [7,8]. Australian health authorities recommend daily supplementation of folic acid three months before conception and for the first three months of pregnancy, to reduce the risk of NTDs [2,3]. Likewise, mandatory iodine fortification was implemented in Australia in 2009, which required the replacement of non-iodised salt with iodised salt for bread making, with the exception of organic breads. Subsequently, the proportion of females of child-bearing age estimated to have inadequate iodine intakes decreased from 60% to 9% [9]. Daily supplementation of iodine is recommended in Australia three months before conception, as well as for pregnancy and lactation to reduce the risk of iodine deficiency and associated negative impacts on early brain and nervous system development [2,3]. Other nutrients (including iron, omega-3 fatty acids, vitamin D, calcium, and vitamin B12) may be recommended to reproductive aged women with a diagnosed deficiency or inadequate dietary intake [2,3].

Despite supplement use becoming a common practice in Australia, little is known of the national occurrence during preconception, or the demographic characteristics of women who use supplements during this time. There are a limited number of studies on dietary supplement use in Australia during preconception, typically defined as three months before conception, with the prevalence varying considerably (29–67%) [10,11,12,13]. The generalizability of the available data is limited with recruitment using convenience sampling from antenatal clinics, and studies collecting data retrospectively (when women are pregnant), increasing the risk of recall bias. 

Therefore, the aim of this study was to report on the number of women taking dietary supplements during preconception in Australia using national data from the Australian Longitudinal Study on Women’s Health (ALSWH), to explore demographic characteristics and to identify the most common nutrients supplemented by women during preconception.

## 2. Materials and Methods 

### 2.1. Data Collection

The current study used self-reported data collected prospectively from the ALSWH. The ALSWH recruited 40,393 women in 1996 across three cohorts; those born in 1973–78 (18–23 years), 1946–51 (45–50 years), and 1921–26 (70–75 years), and more recently, a new cohort born in 1989–95 who were first surveyed in 2013 (aged 18–23 years). When the ALSWH began, women were randomly selected from Australia’s nationalised health-care system, Medicare, with intentional oversampling in rural and remote areas. Ethical approval was obtained by the Human Research Ethics Committees of the University of Newcastle (H-076-0795) prior to baseline data collection in 1996, with written consent provided by participants. Further details on the ALSWH recruitment and cohort profile have been published elsewhere [14,15,16,17].

The present paper examines data from the 1973–78 cohort, who were broadly representative of Australian women the same age at the baseline survey [14]. Paper-based surveys were mailed to participants for Survey 1 in 1996 (*n* = 14,247), Survey 2 in 2000 (*n* = 9688), Survey 3 in 2003 (*n* = 9081), and Survey 4 in 2006 (*n* = 9145). In 2009 (Survey 5, *n* = 8200), 2012 (Survey 6, *n* = 8009), and 2015 (Survey 7, *n* = 7186) participants could opt to complete the survey online or in hard copy. The ALSWH surveys include a broad range of demographic and health behaviour measures, including area of residence, marital status, level of education, parity, smoking status, alcohol use, frequency and intensity of physical activity, weight, height, and income.

### 2.2. Sample

Women were aged 31 to 36 years at the time of completing Survey 5 in 2009. To derive preconception status, the women’s Survey 5 return date and child’s date of birth data from subsequent surveys were used. Data management for the ALSWH involves de-identifying participant information, including child dates of birth. All child dates of birth for a particular month are rounded to the 15th of that month. For example, a child date of birth occurring on 1 June would be rounded to 15 June, as would a child date of birth occurring on 30 June. Following the methods of Gresham et al., ‘preconception’ included women who were 1–6 months before a conception resulting in a birth (i.e., Survey 5 returned 10–15 months before a child’s date of birth recorded at a subsequent survey) [18]. Women with a pregnancy resulting in a miscarriage or termination would not have been identified, however, stillbirths may have been included. 

### 2.3. Supplement Data

At Survey 5, women were asked to write down the names of all their medications, vitamins, supplements or herbal therapies they had taken in the last four weeks, with an option to select ‘none’. Women were excluded from the present analyses if they (i) were not classified as preconception when completing the survey (*n* = 7691) or (ii) were classified as preconception with missing supplement data (*n* = 24).

Women were classified as ‘supplementing’ if at least one dietary supplement was reported, while women who responded ‘none’ were classified as ‘not supplementing’. Participants were not required to specify the dose or quantity of the supplement consumed, nor did some participants specify the brand. Over-the-counter and prescription supplements were included in this study.

Three stages were used to categorise supplements. The first stage included the World Health Organization (WHO) Anatomical Therapeutic Chemical (ATC) classification system with Defined Daily Dose (DDD). This type of coding categorises drugs into different groups according to the organ or system on which they act and their therapeutic, pharmacological, and chemical properties, in addition to the assumed average maintenance dose per day for the drugs main indication in adults [19]. Further details on the ATC/DDD classification system are available elsewhere [19]. The main active ingredients of the formulation for each original medication and supplement were identified and assigned an ATC code. Other medications, herbal preparations, and tonics were excluded for this analysis.

The second stage of supplement classification used the ATC/DDD system as a framework to generate an extensive list of single nutrient (i.e., folate, iron, calcium), combination nutrients (i.e., iron and folate, iodine, and folate) and multiple micronutrient supplement (MMN) (i.e., three or more micronutrients) categories, without therapeutic doses. The final stage of supplement classification grouped the classifications from the second stage if the supplement category included folic acid, iodine, or iron. 

### 2.4. Other Variables

Women reported their country of birth at Survey 1 (baseline) in 1996. More current demographic characteristics including area of residence, marital status, level of education, parity, smoking status, alcohol use, frequency and intensity of physical activity, weight, height, and income were reported at Survey 5 in 2009. Women were also asked at Survey 5 if they had been diagnosed or treated for ‘low iron (iron-deficiency or anaemia)’ in the past three years and if they were ‘trying to become pregnant’.

### 2.5. Statistical Analysis

The characteristics of women who were included in the analysis were compared to the remaining 1973-78 ALSWH cohort. The characteristics of women who were classified as preconception and supplementing were compared to women who were preconception and not supplementing. Data were checked for normality using numerical and graphical methods including the Shapiro–Wilk test and histograms. Means and standard deviations were presented for normally distributed continuous variables and groups were compared using two sample *t*-tests with unequal variance. Proportions were presented for categorical variables, and groups were compared using Pearson chi-square test (*X*^2^) for independence, or in the case of small cell sizes, Fisher’s exact test. A *p* value of ≤ 0.01 was considered statistically significant. All analyses were performed using the statistical software package Stata IC, version 13 (StataCorp, College Station, TX, USA) [20]. 

## 3. Results

**Figure 1:**
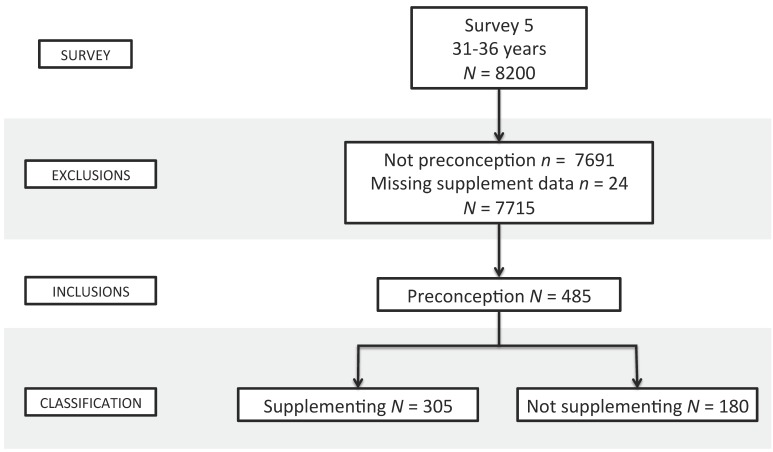
Selection of participants and classification of supplementation status.

A total of 485 preconception women were included in the analysis; 6% of all women who completed Survey 5. The selection of cohort participants eligible for inclusion, including the classification of women supplementing (or not) is presented in Figure 1. 

Table 1 summarises the baseline characteristics (reported in 1996) of women included in the analysis and for those in the remaining 1973–78 ALSWH cohort. Women included were the same age as those excluded (20.6 and 20.8 years, respectively), with the majority from both groups engaging in high levels of physical activity (34.2% vs. 30.1%). At baseline, women included were more likely to be living in urban areas, be single, have no children, and be less likely to smoke or drink alcohol at risky levels. While there was a similar number of women who attained school or high-school education, more women included in the current analysis reported university education (16.4% vs. 10.9%).

Women who reported taking supplements during preconception were more likely to be trying to conceive (48% vs. 23%; *p* ≤ 0.001), have no previous children (35% vs. 21%; *p* ≤ 0.001), and were born outside of Australia (*p* ≤ 0.001) when compared to women who did not supplement (Table 2). There were no statistically significant differences in regards to age, Body Mass Index, education, area of residence, annual household income, marital status, smoking, alcohol intake, or physical activity.

During preconception, 63% of women (*n* = 305) reported taking at least one dietary supplement. Of those women, 57% (*n =* 173) reported taking only one type of supplement, 28% (*n* = 86) reported taking two types of supplements, and 15% (*n* = 46) reported taking three or more. The highest reported number of supplements taken during preconception was six supplements (*n* = 3).

Table 3 reports the most common types of supplements used by women during preconception. MMN supplements were the most common type of supplement (44%), followed by single nutrient supplements (34% of women reporting). The six most commonly reported single nutrient supplements included folic acid (13%), omega-3 fatty acids (11%), vitamin C (7%), B vitamins (4%), iron (3%), and calcium (3%).

**Figure 2:**
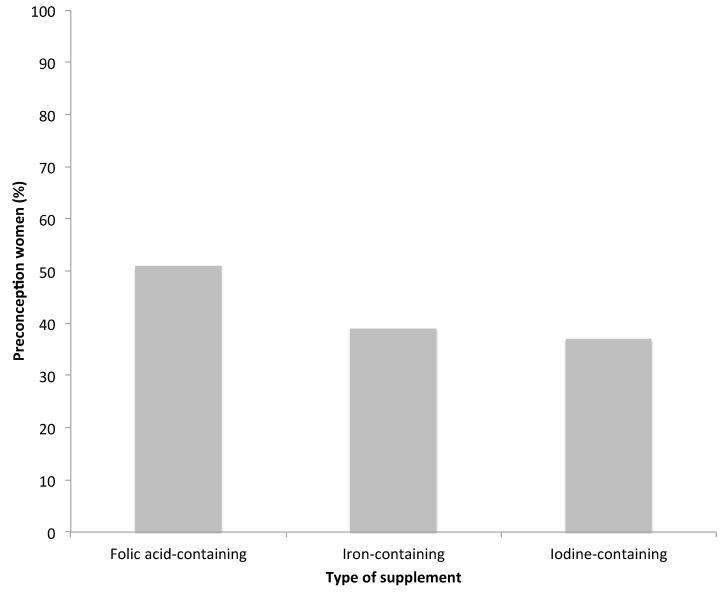
Nutrient-containing dietary supplement use among preconception women aged 31–36 years in 2009 from the Australian Longitudinal Study on Women’s Health. Participants were able to select multiple supplements, therefore numbers do not add to 100%. Nutrients from dietary supplements includes nutrients from both multiple micronutrient and single nutrient supplements.

Approximately half of the women (51%) reported taking a folic acid-containing supplement, 39% an iron-containing supplement, and approximately a third (37%) an iodine-containing supplement during preconception (Figure 2). Of the women taking an iron-containing supplement, 23% reported an iron deficiency, while the majority (70%) did not report a pre-diagnosed iron deficiency and the remaining had missing data.

## 4. Discussion

This study found that just under two thirds (63%) of women aged 31–36 years took one or more dietary supplements during preconception, which broadly captured about one to six months before conception resulting in a live or still-birth. Our study is novel, in that we have analysed data collected prospectively, before the outcome of a planned or unplanned pregnancy was known. Two out of every five women in this study identified that they were ‘trying to conceive’ in the window we defined as preconception, suggesting that supplementation use is high in women of this age generally, rather than because of recommendations for preconception and pregnancy. In our study, if a woman had given birth preterm, they may have completed the survey as early as eight months prior to conception, which could explain the slightly lower rate (40%) of women who indicated that they were actively ‘trying to conceive’, compared to the national average (half of all pregnancies are unplanned) [21]. Previous research on contraceptive use and unintended pregnancy in Australia has identified issues of ambiguity and ambivalence around a woman’s intentions to fall pregnant, which impacts on a woman’s decision to use contraceptives (or not) and the reliability of contraceptives that they use [22]. Women in our study who reported they were not trying to conceive were also less likely to take supplements during preconception, which seems consistent with having some measure of the supplementing behaviours in unplanned pregnancies.

### 4.1. Interpretation

The high rate of supplementation in our study is similar to the findings of two studies conducted in Australia at large hospitals in major cities [10,11]. Women who were supplementing prior to pregnancy were more often trying to conceive, with no previous children and be born overseas, compared to women not supplementing. 

In our study, 13% of women (preconception), reported supplementing with a folic acid-only supplement, increasing to 51% when all folic acid-containing supplements were included. Similar results have been found by other Australian studies reporting on folic acid supplementation; ranging from 27–61% [11,13,23]. 

Supplementation of iodine was low among women before conception, with approximately a third of women (37%) supplementing with an iodine containing preparation. Our findings are lower than rates reported by a recent study (2016) that collected data from a national online cohort (*n* = 455) and South Australian public maternity hospital cohort (*n* = 402) of pregnant women, where approximately 50% of women reported supplementing with an iodine containing preparation during preconception [11]. This increase may be due to the higher rate of planned pregnancies (74%) reported, in addition to an increasing awareness of supplementation during pregnancy overtime and an increased risk of recall bias associated with retrospective data collection. A higher supplementation rate found by studies in more recent years may be explained by the Australian iodine supplementation recommendations for pregnant women and women considering pregnancy released in 2010 [24], following our supplementation data collection in 2009. Given the low rates of folic acid and iodine supplementation, further research is needed to quantify total oral intake of such nutrients (inclusive of foods containing mandatory fortification of folate and iodine) and whether women are meeting the requirements through diet alone. The barriers to such supplementation, and ongoing education of reproductive aged women about the importance of folic acid and iodine supplementation may be required, if women are not meeting these requirements through diet alone.

Our findings of MMN supplementation among preconception women (44%) is consistent with the findings of a recent study conducted in Sydney where pregnant women were recruited from antenatal clinics at two tertiary teaching hospitals (*n* = 589) [10]. The most frequent MMN supplements reported in our study were among the top market leaders of Australian pregnancy-specific supplements, consistent with other Australian studies’ findings [12,25]. These market leader preparations contain a range of nutrients, varying in their dosage of nutrients, and not aligning with the Australian recommendations for supplementation during preconception. One product provides an excess of 300 µg of folic acid and 70 µg of iodine, whilst another provides the recommended amounts for both nutrients [2,3]. These market leader supplements also provide nutrients (including iron, calcium, omega-3 fatty acids, vitamin D, and B vitamins) that are not currently recommended without a confirmed deficiency or low dietary intake, potentially increasing the risk of harm caused by an excessive dietary intake and/or high levels of such nutrients in the body. 

Supplementing with iron is not routinely recommended to women in Australia during preconception, due to a lack of evidence for its benefit and increased risk of adverse outcomes during pregnancy [2,26]. Despite the recommendations, our findings show the rate of iron-containing supplementation to be higher than that for iodine-containing (which should be routinely recommended for all women considering pregnancy, during preconception, pregnancy, and lactation). In addition, approximately 70% of women who were supplementing with an iron-containing supplement did not self-report an iron deficiency. There is potential that some of these women were unaware they were low in iron and therefore undiagnosed, however, further research is warranted to determine the potential short and long-term effects of iron supplementation among women without diagnosed deficiencies prior to conception and during pregnancy. 

### 4.2. Implications for Practice and Research

With lower than previously reported rates of folic acid and iodine supplementation during preconception found in this study, alongside the fact that less than half of all pregnancies are being planned, the importance of mandatory fortification of such nutrients to provide adequate reach to the preconception population is highlighted. 

General practitioners are generally one of the first health professionals a woman consults to confirm that she is pregnant. This initial consultation would be of no benefit in providing information on dietary supplements during the preconception period, particularly for the reduced risks of NTDs and impaired cognitive development associated with folic acid and iodine supplement use, respectively. However, this consult could be used to identify women with or at risk of nutrient deficiencies, where strategies should be put into place to improve dietary intake and quality during pregnancy, as well as highlighting the importance of at-risk nutrients for subsequent pregnancies.

Further studies are required to quantify total nutrient intake during preconception, while further investigations into supplement use prior to conception and adverse pregnancy and birth outcomes are needed.

### 4.3. Strengths and Limitations

This study is the first study, worldwide, to report on dietary supplement use during preconception using data collected prospectively. The retention rate for Survey 5 in 2009 was >55% of the initial study sample. Research has shown that women responding at all ALSWH survey waves (Surveys 1–4) are more educated, less likely to smoke and have children than those women responding to some of the surveys. However, while there is a need to consider the potential for bias due to attrition, the identified biases are insufficient to preclude meaningful longitudinal analyses in this cohort of women (1973–1978) [27].

Due to de-identification of participant information, specifically child date of birth data, which is rounded to the 15th day of their birth month, there may be potential for misclassification error according to preconception status using the child’s date of birth and survey return date. This would only occur for women who delivered preterm and returned their survey within the number of months they delivered preterm: for example, a woman who delivered at seven months (i.e., 32 weeks gestation) and returned her survey two months preconception, would not have been included in our study, as this survey would fall outside the 10–15 month window. Furthermore, our case definition relies on a date of birth being recorded, so any pregnancy that resulted in a miscarriage or termination would not have been identified, however stillbirth may have been included.

The current study included women aged 31–36 years. In 2009–2010, the median age of women giving birth in Australia was approximately 31 years [28], highlighting that our findings may be broadly generalizable to women of the same age in the Australian population. 

Gresham et al. and Hure et al. previously conducted agreement studies of women in the 1973–1978 cohort, demonstrating high agreement (≥87%) between self-reported ALSWH and administrative data for the adverse pregnancy outcomes gestational hypertension, gestational diabetes mellitus, preterm birth, low birth weight [29], and stillbirth [30], offering a high degree of confidence in the accuracy of self-report of women in the ALSWH. 

Data on nutrient amounts and frequency of supplementation was unavailable at Survey 5, as women were not asked to provide this information, and as a result total nutrient intakes from dietary supplements and adherence to the recommendations for daily dosage and compliance of supplement regimes were unable to be reported.

Data for this study were collected in 2009, following the development of the Royal Australian and New Zealand Colleague of Obstetricians and Gynaecologists (RANZCOG) ‘Vitamin and mineral supplementation and pregnancy’ statement in 2008 [3]. Over time, these recommendations have been revised and, in 2011, iodine supplementation was included as a recommendation. During this time there was a reduction in the supplemental dose for folate by 100 μg/day (500 to 400 μg/day), which has not impacted on the reported findings in this study, as supplement intake was not quantified by dosage. Our study found lower rates of iodine supplementation, which may be a result of iodine not being recommended as a standard supplementation for women who were trying to conceive in 2009.

## 5. Conclusions

The current study is the first study to report on dietary supplement use during preconception in Australia using data collected prospectively from preconception women. The findings suggest that the majority of Australian women take at least one dietary supplement during preconception. Women are frequently taking supplements containing multiple nutrients, of which some of the included nutrients are recommended only to women with diagnosed deficiencies or low dietary intakes. The low supplementation rates of folic acid and iodine warrant further public health interventions to increase awareness of their importance, while further research is needed to determine the role of dietary supplementation during preconception, evaluating short and long term pregnancy outcomes and measuring other factors such as total nutrient intakes and diet quality.

## Figures and Tables

**Table 1:** Baseline characteristics † for the young cohort of the Australian Longitudinal Study on Women’s Health 1973–1978 according to inclusion (*n* = 485) or not in the present study (*n* = 13,762).

	Included in the Study *N* = 485			Not Included in the Study *N* = 13,762		
Characteristics	*n*	Mean	SD	*n*	Mean	SD
**Age**	485	20.6	1.4	13,762	20.8	1.5
	*n*	%		*n*	%	
**Australian Residence**						
Urban	313	64.5		7556	54.9	
Rural	161	33.2		5660	41.1	
Remote	11	2.3		546	4	
Missing	0	-		0	-	
**Marital Status**						
Married/Defacto	61	12.7		3132	22.9	
Separated/Divorced	1	0.2		128	0.9	
Single	419	87.1		10,431	76.2	
Missing	4	-		66	-	
**Parity**						
None	475	98.3		12,137	89.7	
One	5	1.1		1023	7.5	
Two or more	3	0.6		374	2.8	
Missing	2	-		228	-	
**Highest educational level**						
No formal education	5	1		403	3	
School or higher school certificate	323	66.9		9296	67.9	
Trade or Diploma	76	15.7		2487	18.2	
University or Higher university degree	79	16.4		1497	10.9	
Missing	2	-		79	-	
**Physical activity**						
Nil/sedentary	53	11		2085	15.3	
Low	141	29.3		3941	28.8	
Moderate	123	25.5		3523	25.8	
High	165	34.2		4122	30.1	
Missing	3	-		91	-	
**Smoking**						
Current smoker	118	25.2		4303	32.7	
Non-smoker	350	74.8		8858	67.3	
Missing	17	-		601	-	
**Alcohol Intake**						
Non-drinker	31	6.5		1223	9	
Low risk/rarely drinks	426	88.9		11,626	85.4	
High risk/risky drinker	22	4.6		760	5.6	
Missing	6	-		153	-	

† Participant characteristics were taken from the baseline survey; SD, Standard Deviation; -, Percent is not calculated for missing values.

**Table 2:** Demographic characteristics of women aged 31–36 years in 2009 from the Australian Longitudinal Study on Women’s Health who were supplementing and not supplementing during preconception.

	Supplementing			Not Supplementing			
	*N* = 305			*N* = 180			
Characteristics	*n*	Mean	SD	*n*	Mean	SD	*p* Value
**Age**	305	33.5	1.4	180	33.5	1.4	0.887
	*n*	%		*n*	%		
**Country of Birth**							
Australia	277	91.4		172	96.6		0.009
Outside of Australia	26	8.6		6	3.4		
Missing	2	-		2	-		
**Body Mass Index**							
Underweight	6	2		3	1.7		0.568
Healthy Weight	183	60.4		98	54.7		
Overweight	76	25.1		49	27.4		
Obese	38	12.5		29	16.2		
Missing	2	-		1	-		
**Australian Residence**							
Urban	203	67.7		107	62.2		0.179
Rural	90	30		56	32.6		
Remote	7	2.3		9	5.2		
Missing	5	-		8	-		
**Marital Status**							
Married/Defacto	289	95.1		173	96.1		0.939
Separated/Divorced	8	2.6		3	1.7		
Single	7	2.3		4	2.2		
Missing	1	-		0	-		
**Parity**							
None	107	36.1		37	20.6		<0.001
One	139	47		85	47.2		
Two or more	50	16.9		58	32.2		
Missing	0	-		0	-		
**Highest educational level**							
No formal education	2	0.7		2	1.1		0.727
School or higher school certificate	36	12		23	13.1		
Trade or Diploma	62	20.6		30	17.1		
University or higher university degree	201	66.8		120	68.6		
Missing	4	-		5	-		
**Annual Household Income**							
No income	2	0.7		1	0.6		0.292
$1–$36,999	12	4		5	2.8		
$37,000–$129,999	169	55.8		118	65.9		
$130,000 or more	98	32.3		45	25.1		
Don’t know/Don’t want to answer	22	7.3		10	5.6		
Missing	2	-		1	-		
**Physical activity**							
Nil/sedentary	25	8.4		26	14.9		0.138
Low	117	39.5		65	37.4		
Moderate	85	28.7		41	23.6		
High	69	23.3		42	24.1		
Missing	9	-		6	-		
**Smoking**							
Daily	6	2		11	6.1		0.042
At least weekly (not daily)	3	1		5	2.8		
Less often than weekly	8	2.6		5	2.8		
Not at all	288	94.4		159	88.3		
Missing	0	-		0	-		
**Alcohol Intake**							
Non-drinker	28	9.2		23	12.8		0.435
Low risk/rarely drinks	260	85.5		148	82.7		
High risk/risky drinker	16	5.3		8	4.5		
Missing	1	-		1	-		
**Trying to conceive**							
Yes	149	48.4		42	23.5		<0.001
No	159	51.6		137	76.5		
Missing	0	-		1	-		

SD, Standard Deviation; -, Percent is not calculated for missing values.

**Table 3:** Rates of multiple and single micronutrient supplement use among preconception women aged 31–36 years in 2009 from the Australian Longitudinal Study on Women’s Health.

	Preconception *N* = 485	
Supplement	*n*	%
**Multiple Micronutrient**	212	43.7
**Single nutrient**	167	34.4
Folic acid	62	12.8
Omega-3/Fish oil	52	10.7
Vitamin C	33	6.8
B Vitamins	18	3.7
Iron	15	3.1
Calcium	14	2.9

Participants were able to select multiple supplements, therefore numbers are not mutually exclusive.

## References

[1] World Health Organization (2016). WHO Recommendations on Antenatal Care for a Positive Pregnancy Experience.

[2] Australian Health Ministers’ Advisory Council (2012). Clinical Practice Guidelines: Anetnatal Care—Module 1.

[3] Womens Health Committee (2015). Vitamin and Mineral Supplementation and Pregnancy.

[4] Czeizel A.E., Dudas I. (1992). Prevention of the first occurrence of neural-tube defects by periconceptional vitamin supplementation. N. Engl. J. Med..

[5] De-Regil L.M., Pena-Rosas J.P., Fernandez-Gaxiola A.C., Rayco-Solon P. (2015). Effects and safety of periconceptional oral folate supplementation for preventing birth defects. Cochrane Database Syst. Rev..

[6] Marieb E.N., Hoehn K. (2015). Human Anatomy and Physiology.

[7] Hilder L., National Perinatal Epidemiology and Statistics Unit (2016). Neural Tube Defects in Australia 2007–2011: Before and after Implementation of the Mandatory Folic Acid Fortification Standard.

[8] Brown R.D., Langshaw M.R., Uhr E.J., Gibson J.N., Joshua D.E. (2011). The impact of mandatory fortification of flour with folic acid on the blood folate levels of an Australian population. Med. J. Aust..

[9] Food Standards Australia New Zealand (2016). Monitoring the Australian Population’s Intake of Dietary Iodine before and after Mandatory Fortification.

[10] Shand A.W., Walls M., Chatterjee R., Nassar N., Khambalia A.Z. (2016). Dietary vitamin, mineral and herbal supplement use: A cross-sectional survey of before and during pregnancy use in Sydney, Australia. Aust. N. Z. J. Obstet. Gynaecol..

[11] Malek L., Umberger W., Makrides M., Zhou S.J. (2016). Poor adherence to folic acid and iodine supplement recommendations in preconception and pregnancy: A cross-sectional analysis. Aust. N. Z. J. Public Health.

[12] El-Mani S., Charlton K.E., Flood V., Mullan J. (2014). Limited knowledge about folic acid and iodine nutrition in pregnant women reflected in supplementation practices. Nutr. Diet..

[13] Lucas C.J., Charlton K.E., Brown L., Brock E., Cummins L. (2014). Antenatal shared care: Are pregnant women being adequately informed about iodine and nutritional supplementation?. Aust. N. Z. J. Obstet. Gynaecol..

[14] Brown W.J., Bryson L., Byles J.E., Dobson A.J., Lee C., Mishra G., Schofield M. (1999). Women’s health Australia: Recruitment for a national longitudinal cohort study. Women Health.

[15] Lee C., Dobson A.J., Brown W.J., Bryson L., Byles J., Warner-Smith P., Young A.F. (2005). Cohort profile: The Australian longitudinal study on women’s health. Int. J. Epidemiol..

[16] Brown W., Lois B., Byles J., Dobson A., Manderson L., Schofield M., Williams G. (1996). Women’s health Australia: Establishment of the australian longitudinal study on women’s health. J. Women Health.

[17] Dobson A.J., Hockey R., Brown W.J., Byles J.E., Loxton D.J., McLaughlin D., Tooth L.R., Mishra G.D. (2015). Cohort profile update: Australian longitudinal study on women’s health. Int. J. Epidemiol..

[18] Gresham E., Collins C.E., Mishra G.D., Byles J.E., Hure A.J. (2016). Diet quality before or during pregnancy and the relationship with pregnancy and birth outcomes: The Australian longitudinal study on women’s health. Public Health Nutr..

[19] WHO Collaborating Centre for Drug Statistics Methodology (2012). Guidelines for ATC Classification and DDD Assignment 2013.

[20] StataCorp (2013). Stata Statistical Software: Release 13.

[21] Children by Choice Association (2013). Fact Sheet: Unplanned Pregnancy in Australia.

[22] Coombe J., Harris M.L., Wigginton B., Lucke J., Loxton D. (2016). Contraceptive use at the time of unintended pregnancy: Findings from the contraceptive use, pregnancy intention and decisions study. Aust. Fam. Phys..

[23] Watson L.F., Brown S.J., Davey M.A. (2006). Use of periconceptional folic acid supplements in Victoria and New South Wales, Australia. Aust. N. Z. J. Public Health.

[24] National Health and Medical Research Council (2010). Iodine Supplementation for Pregnant and Breastfeeding Women.

[25] Conlin M.L., MacLennan A.H., Broadbent J.L. (2006). Inadequate compliance with periconceptional folic acid supplementation in South Australia. Aust. N. Z. J. Obstet. Gynaecol..

[26] Pena-Rosas J.P., De-Regil L.M., Garcia-Casal M.N., Dowswell T. (2015). Daily oral iron supplementation during pregnancy. Cochrane Database Syst. Rev..

[27] Powers J., Loxton D. (2010). The impact of attrition in an 11-year prospective longitudinal study of younger women. Ann. Epidemiol..

[28] Australian Bureau of Statistics (2016). 3301.0—Births, Australia, 2015.

[29] Gresham E., Forder P., Chojenta C.L., Byles J.E., Loxton D.J., Hure A.J. (2015). Agreement between self-reported perinatal outcomes and administrative data in New South Wales, Australia. BMC Pregnancy Childbirth.

[30] Hure A.J., Chojenta C.L., Powers J.R., Byles J.E., Loxton D. (2015). Validity and reliability of stillbirth data using linked self-reported and administrative datasets. J. Epidemiol..

